# Effects of different intraoperative warming strategies on hypothermia and postoperative coagulation function in severely injured emergency surgery patients

**DOI:** 10.3389/fmed.2026.1755372

**Published:** 2026-05-05

**Authors:** Lei Zhang, Weiming Qian

**Affiliations:** Department of Nursing, The Second Affiliated Hospital of Zhejiang University School of Medicine, Hangzhou, Zhejiang, China

**Keywords:** coagulation function, emergency surgery, forced-air warming, perioperative hypothermia, severely injured, temperature management

## Abstract

**Objective:**

To compare the effects of routine and enhanced intraoperative warming strategies on perioperative hypothermia, coagulation function, and length of hospital stay in severely injured emergency surgery patients.

**Methods:**

A retrospective cohort study was conducted on 134 severely injured emergency surgery patients (January 2023–December 2024), divided into a routine warming group (*n* = 87) and an enhanced warming group (*n* = 47). The primary outcome was hypothermia incidence at 1 h postoperative (T3), defined as core body temperature < 36.0 °C. Secondary outcomes included activated partial thromboplastin time (APTT) and prothrombin time percentage (PT%) at 12–24 h postoperative (C3), and length of hospital stay. Core body temperature was monitored at preoperative (T1), end of surgery (T2), and T3. Coagulation parameters were measured at 1 h preoperative (C1), 2–3 h postoperative (C2), and C3. Multivariable regression analyses adjusted for age, sex, injury severity score, operative duration, intraoperative blood loss, and red blood cell transfusion volume.

**Results:**

Baseline characteristics were comparable between groups (all *p* > 0.05). The enhanced warming group had significantly higher core body temperature at T2 and T3 (*p* < 0.05), with a lower hypothermia incidence at T3 (*p* < 0.05). After multivariable adjustment, enhanced warming remained independently associated with reduced hypothermia risk at T3 (adjusted OR = 0.37, 95% CI 0.16–0.84, *p* = 0.018) and lower APTT at C3 (*β* = −4.62, *p* = 0.033), while the PT% difference did not retain significance after adjustment (*p* = 0.076). Fibrinogen was significantly lower in the enhanced warming group at all time points including baseline, indicating a pre-existing difference unrelated to the intervention. The enhanced warming group had a significantly shorter length of hospital stay after adjustment (*β* = −3.42 days, *p* = 0.036).

**Conclusion:**

Enhanced intraoperative warming was associated with lower postoperative hypothermia incidence, reduced APTT, and shorter hospital stay after confounder adjustment. Prospective trials with baseline coagulation stratification are needed to confirm these findings.

## 1 Introduction

The clinical consequences of hypothermia in trauma patients are particularly severe (1). Inadvertent perioperative hypothermia (a phenomenon where the core body temperature is lower than 36 °C and is unrelated to medically purposeful hypothermia) has an incidence rate of 10–80% among general surgical patients, and reaches 67% in patients with severe trauma (2–4). The development of hypothermia in trauma surgery is attributed to a combination of factors, including environmental exposure, anesthesia-induced vasodilation, extensive surgical exposure, and infusion of cold intravenous fluids and blood products (5).

The pathophysiological consequences of perioperative hypothermia extend far beyond patient discomfort, triggering a series of cascading adverse reactions that significantly impact patient outcomes. Hypothermia impairs plasma coagulation function—as coagulation factors and platelet function require optimal temperature ranges to operate normally, the activity of related enzymes and the efficiency of the coagulation cascade significantly decrease with falling body temperature (6). This temperature-dependent coagulopathy manifests as increased bleeding tendency, with studies showing that mild hypothermia is associated with increased bleeding risk and transfusion requirements (5). Additionally, hypothermic patients exhibit impaired immune function through suppression of monocyte HLA-DR expression, reduced leukocyte migration and phagocytic capacity, and effects on cytokine production, leading to increased surgical site infection rates (7, 8). Hypothermia also causes cardiovascular complications (such as arrhythmias and myocardial ischemia), affects drug metabolism and clearance, delays wound healing processes, and ultimately leads to prolonged hospital stays and increased medical costs (6).

Current literature predominantly focuses on elective surgical populations, with limited evidence addressing optimal warming strategies specifically for severely injured emergency surgery patients. This population faces unique challenges including compromised physiological reserves, massive bleeding, and urgent surgical requirements that may render standard warming protocols insufficient. Recent guidelines recommend active warming strategies over passive measures, including forced-air warming systems, environmental temperature control, and warmed intravenous fluids (9). Studies demonstrate that multimodal approaches combining these techniques may achieve superior temperature maintenance compared to single interventions (10, 11). However, the optimal combination and implementation of these strategies specifically for trauma patients undergoing emergency surgery remains unclear.

This study aimed to compare the effects of routine and enhanced warming strategies on perioperative hypothermia, coagulation parameters, and length of hospital stay in severely injured patients undergoing emergency surgery. A retrospective cohort design was used, with multivariable regression analysis to adjust for potential confounders including operative duration, intraoperative blood loss, and transfusion volume.

## 2 Methods

### 2.1 Study design

**Figure 1:**
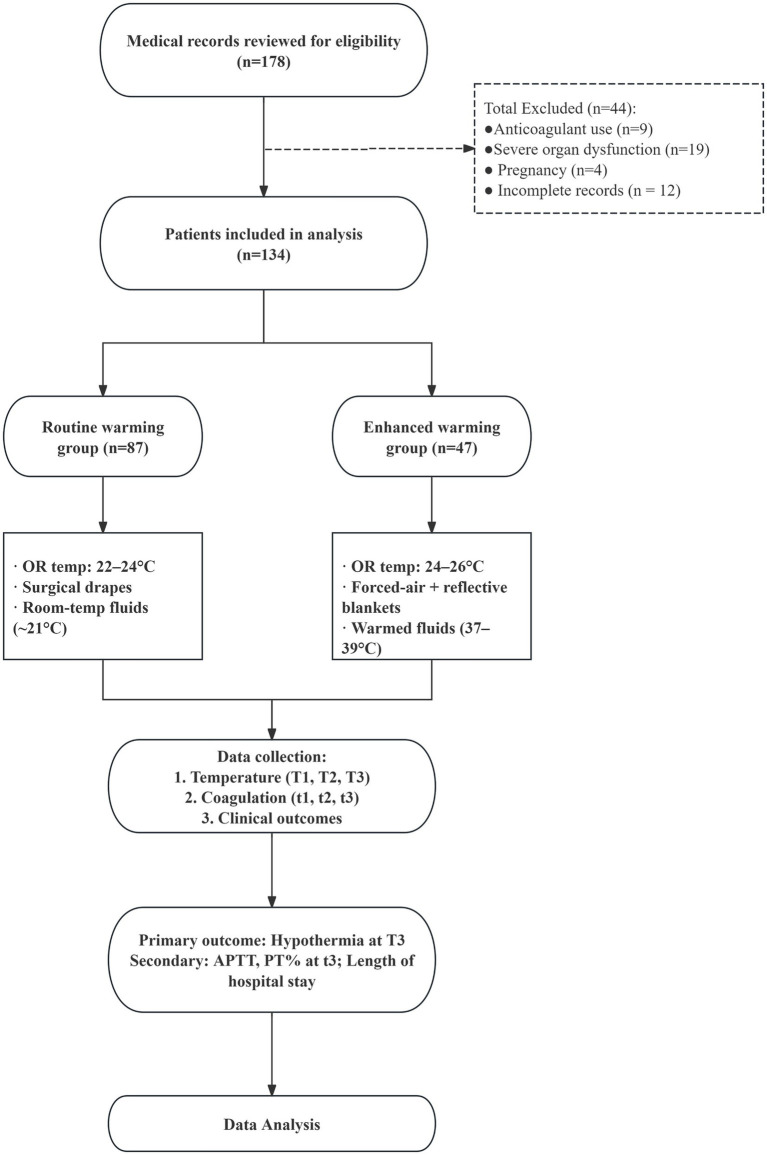
Flow chart of this study.

This study adopted a single-center retrospective cohort design, aiming to evaluate the impact of different warming strategies on perioperative temperature management and coagulation function in patients undergoing emergency surgery for severe trauma. The study spanned from January 2023 to December 2024, with a total of 134 patients who met the inclusion criteria and underwent emergency surgery for severe trauma enrolled. Based on natural grouping according to changes in clinical practice, the patients were divided into the routine warming group (*n* = 87) and the enhanced warming group (*n* = 47). The routine warming protocol was the standard of care from January 1, 2023 to May 31, 2023, and the enhanced warming protocol was implemented as a practice change from June 1, 2023 onward. This study was conducted in accordance with the Declaration of Helsinki and has been approved by the hospital ethics committee (Approval No: 2025 Lun Shen Yan No. 1634). As the study was based on retrospective analysis of existing clinical data, the ethics committee granted a waiver of prospective informed consent. This study is reported in accordance with the Strengthening the Reporting of Observational Studies in Epidemiology (STROBE) guideline for cohort studies. The study procedure is outlined in Figure 1.

### 2.2 Inclusion and exclusion criteria

#### 2.2.1 Inclusion criteria

Patient inclusion was based on internationally recognized severe trauma criteria and surgical indications: (1) Adult patients aged 18–70 years, meeting the population with relatively complete physiological reserve function suitable for surgery (12); (2) Injury Severity Score (ISS) ≥ 16 points (12, 13); and (3) Requiring emergency surgical treatment (4).

#### 2.2.2 Exclusion criteria

To ensure accuracy and comparability of study results, the following patients were excluded: (1) Patients with severe cardiac, hepatic, or renal dysfunction; (2) Patients taking anticoagulants such as warfarin or direct oral anticoagulants (14); (3) Pregnant patients; and (4) Patients with incomplete data.

### 2.3 Warming methods

#### 2.3.1 Routine warming group

The routine warming group received the following warming strategies: (1) Operating room ambient temperature set at 22–24 °C; (2) Routine surgical drape coverage, maintaining body temperature by reducing radiation and convective heat loss; and (3) Intraoperative infusion of room-temperature fluids (approximately 21 °C) (15).

#### 2.3.2 Enhanced warming group

The enhanced warming group received the following warming strategies based on the latest Association of periOperative Registered Nurses guidelines: (1) Operating room ambient temperature raised to 24–26 °C; (2) Forced-air warming blankets applied in combination with reflective warming blankets (10); and (3) Intraoperative infusion of intravenous fluids warmed to 37–39 °C (11).

### 2.4 Outcome collection

Core body temperature was recorded at three time points: preoperative baseline (T1), end of surgery (T2), and 1 h postoperative (T3). Coagulation parameters were measured at 1 h preoperative (C1), 2–3 h postoperative (C2), and 12–24 h postoperative (C3). The primary outcome was the incidence of hypothermia at T3, defined as core body temperature <36.0 °C. Secondary outcomes included coagulation parameters (PT%, APTT) at C3 and length of hospital stay. A between-group temperature difference of 0.5 °C was considered clinically meaningful based on prior literature (16).

#### 2.4.1 Body temperature monitoring

**Figure 2:**
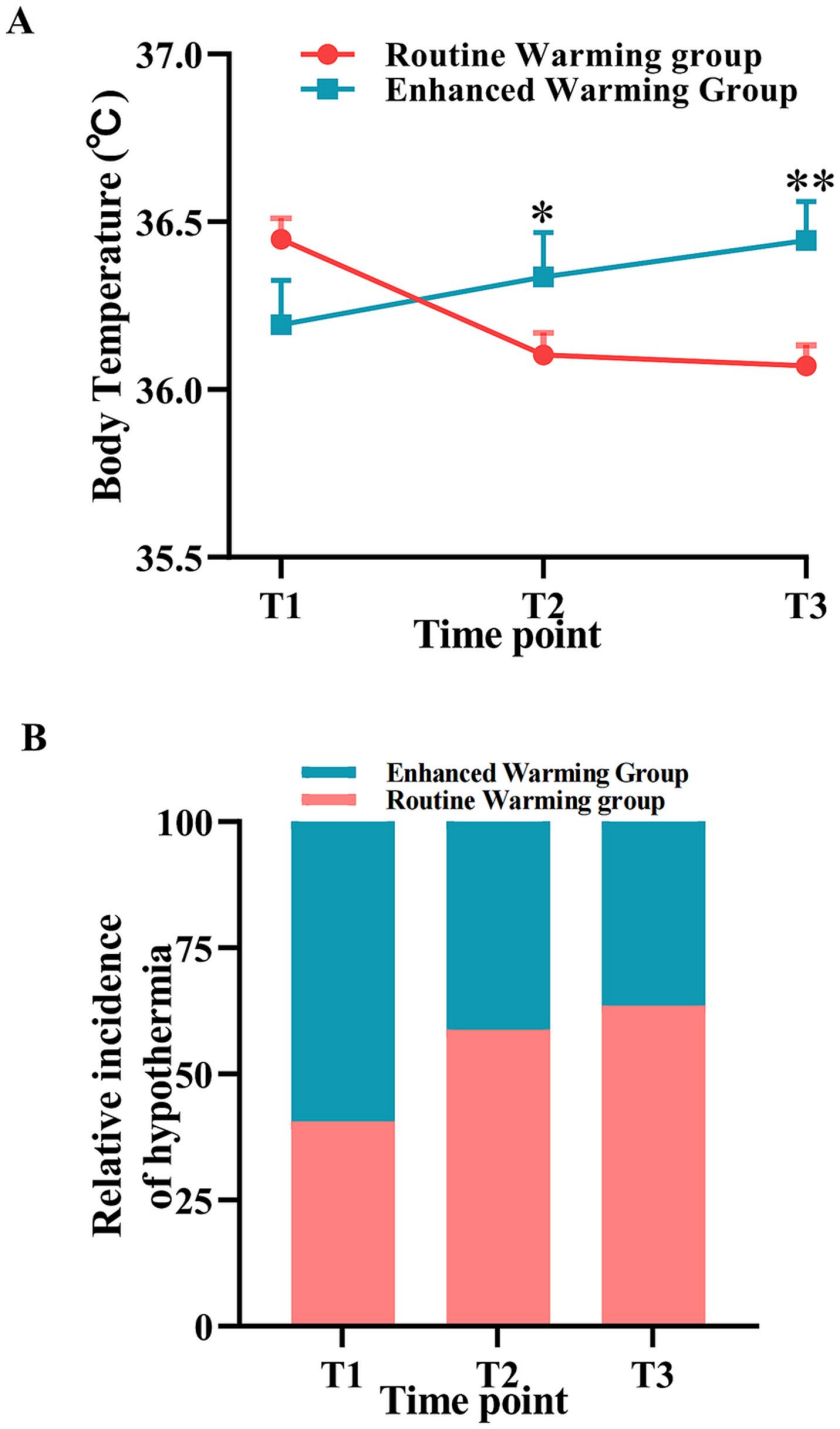
Comparison of body temperature changes and hypothermia incidence at different time points. **(A)** Core body temperature comparison between the enhanced warming group (blue line) and the routine warming group (red line) at preoperative (T1), end of surgery (T2), and 1 h postoperative (T3) time points. **(B)** Incidence hypothermia comparison between the enhanced warming group (blue line) and the routine warming group (red line) at T1, T2, and T3. Data expressed as M (Q1–Q3) or *n* (%), * *p* < 0.05, ** *p* < 0.01.

Core body temperature was monitored with esophageal or nasopharyngeal temperature probes (accuracy ±0.1 °C and response time <30 s). Probes were placed at the lower third of the esophagus (35–40 cm from the incisors), and bladder temperature served as an alternative. Probes were zero-calibrated daily against a certified reference thermometer, and temperature readings at each time point were recorded by ward nurses. Body temperature were classified under the following standard (17): normal (body temperature 36.0–37.5 °C) mild hypothermia (body temperature 35.0–35.9 °C); moderate hypothermia (body temperature 34.0–34.9 °C); severe hypothermia (body temperature <34.0 °C). The incidence of hypothermia at each time point was calculated as the proportion of patients with core temperature <36.0 °C (Figure 2).

#### 2.4.2 Coagulation parameters

Blood samples were collected at 1 h preoperative (C1), 2–3 h postoperative (C2), and 12–24 h postoperative (C3) from a central or peripheral venous catheter into 3.2% sodium citrate tube. Samples were centrifuged within 30 min to separate plasma, which was stored at −80 °C and analyzed in batches. Testing was performed using an ACL TOP 750 automatic coagulation analyzer (Werfen, Barcelona, Spain); fibrinogen was measured by Clauss method and D-dimer by immunoturbidimetry, both using manufacturer-supplied reagents. Daily quality control was performed with normal and abnormal control samples. Reference ranges were: prothrombin time (PT) 11.0–13.0 s; prothrombin time percentage (PT%) 80–120%; international normalized ratio (INR) 0.8–1.2; activated partial thromboplastin time (APTT) 26.0–36.0 s; thrombin time (TT) 14.0–21.0 s; fibrinogen 2.0–4.0 g/L; D-dimer <500 μg/L. Coagulation dysfunction was defined as PT or APTT prolongation >1.5 times the upper limit of normal, and hypofibrinogenemia as fibrinogen <1.5 g/L. Samples showing hemolysis, clotting, or insufficient volume were recollected (18). All data were extracted from the hospital electronic medical record system by two investigators independently, and discrepancies were resolved by reviewing the original records. Cases with more than 20% missing values were excluded.

#### 2.4.3 Clinical outcomes

Length of hospital stay was calculated from the date of surgery to the date of discharge. Postoperative complications were defined as the occurrence of any of the following during hospitalization: surgical site infection, pneumonia, urinary tract infection, venous thromboembolism, reoperation for bleeding, organ dysfunction, or intensive care unit readmission.

### 2.5 Statistical analysis

Data were analyzed with SPSS 26.0 (IBM, Armonk, NY, United States), and figures were generated with GraphPad Prism 9.0 (GraphPad Software, San Diego, CA, United States). The normality of continuous variables was assessed using the Shapiro–Wilk test. Variables with normal distribution were expressed as mean ± standard deviation “(x̄±s)”; non-normally distributed variables were expressed as median (interquartile range, IQR) [M (Q1, Q3)]. Between-group comparisons were performed using independent sample *t*-test for normally distributed data or Mann–Whitney U test for non-normally distributed data. Intra-group comparisons between time points used the Wilcoxon signed-rank test. Categorical variables were expressed as *n*(%) and compared using the *χ*^2^ test or Fisher’s exact test when expected cell counts were less than 5. Ordinal variables (such as surgical classification level) were compared using Ridit analysis.

To adjust for potential confounding, multivariable logistic regression was used for the primary outcome (hypothermia at T3), and multivariable linear regression was used for secondary outcomes (APTT and PT% at C3, length of hospital stay). Covariates were selected based on clinical relevance and included age, sex, ISS, operative duration, intraoperative blood loss, and red blood cell transfusion volume. Model fit for logistic regression was assessed using the Hosmer-Lemeshow goodness-of-fit test; assumptions of linearity and homoscedasticity were checked for linear regression models. Results of regression analyses were reported as adjusted odds ratios (aOR) or unstandardized regression coefficients (*β*) with 95% confidence intervals (CI). *p* < 0.05 was considered statistically significant.

## 3 Results

### 3.1 General data comparison

A total of 134 patients were included, with 87 in the routine warming group and 47 in the enhanced warming group. Baseline demographic, injury, and intraoperative characteristics are presented in Table 1. No statistically significant differences were observed between the two groups in age, height, weight, sex distribution, ISS, trauma type, surgical classification level, anesthesia type, operative duration, intraoperative blood loss, intraoperative fluid volume, or transfusion volumes (all *p* > 0.05), indicating satisfactory comparability between the two groups at baseline. The majority of patients in both groups underwent general anesthesia (90.80% vs. 93.62%). The median operative duration was 178 min in the routine warming group and 192 min in the enhanced warming group. Median intraoperative blood loss was 500 mL and 600 mL, respectively.

**Table 1:** Demographics and clinical characteristics.

Characteristic	Routine warming group (*n* = 87)	Enhanced warming group (*n* = 47)	t/U/χ^2^	*p*-value
Demographics				
Age (years)^a^	54 (42, 63)	52 (40, 65)	U = 1928	0.512
Height (cm)^b^	168.5 ± 8.3	169.2 ± 7.9	t = 0.487	0.627
Weight (kg)^a^	67 (58, 76)	68 (57, 78)	U = 1965	0.718
Sex, *n* (%)			χ^2^ = 0.029	0.864
Male	57 (65.52)	30 (63.83)		
Female	30 (34.48)	17 (36.17)		
Injury and surgical characteristics				
ISS^a^	27 (21, 36)	29 (22, 38)	U = 1798	0.198
Trauma type, *n* (%)			χ^2^ = 2.156	0.707
Abdominal	27 (31.03)	17 (36.17)		
Thoracic	16 (18.39)	9 (19.15)		
Craniocerebral	20 (22.99)	7 (14.89)		
Orthopedic/Spinal	14 (16.09)	8 (17.02)		
Multiple sites	10 (11.49)	6 (12.77)		
Surgical classification level, *n* (%)			Z = 0.985	0.325
I	4 (4.60)	1 (2.13)		
II	12 (13.79)	6 (12.77)		
III	41 (47.13)	19 (40.43)		
IV	30 (34.48)	21 (44.68)		
Anesthesia and intraoperative variables				
*Anesthesia type, n (%)*			–	0.549^c^
General anesthesia	79 (90.80)	44 (93.62)		
Combined/Regional	8 (9.20)	3 (6.38)		
Operative duration (min)^a^	178 (128, 252)	192 (135, 268)	U = 1821	0.241
Intraoperative blood loss (ml)^a^	500 (200, 1,000)	600 (250, 1,150)	U = 1789	0.182
Intraoperative fluid volume (ml)^a^	2,100 (1,500, 2,850)	2,300 (1,550, 3,200)	U = 1762	0.138
RBC transfusion (U)^a^	4 (0, 8)	4 (2, 10)	U = 1842	0.289
Plasma transfusion (ml)^a^	400 (0, 800)	400 (0, 1,000)	U = 1867	0.331

a Presented as median (Q1, Q3), compared using Mann–Whitney U test.

b Presented as mean ± standard deviation, compared using independent-sample *t*-test.

c Fisher’s exact test (expected cell count <5).

ISS, Injury Severity Score; RBC, red blood cell.

### 3.2 Body temperature changes and hypothermia incidence comparison

Body temperature changes and hypothermia incidence comparison results showed no significant difference in body temperature between the two groups at T1 time point (*p* > 0.05). At T2 time point, the enhanced warming group had significantly higher body temperature than the routine warming group (*p* < 0.05), with the difference being more significant at T3 time point (*p* < 0.01). In intra-group comparisons, the enhanced warming group showed significantly increased body temperature at T2 and T3 time points compared to preoperative (*p* < 0.001), while the routine warming group showed significantly decreased body temperature at T3 time point compared to preoperative (*p* < 0.05). Regarding hypothermia incidence, there were no significant differences between groups at T1 and T2 time points (*p* > 0.05), but significant differences existed at T3 time point (*p* < 0.05). Compared with the T1 time point, the incidence of hypothermia in the routine warming group increased significantly, rising from 21.84 to 48.28% (*p* < 0.001); in contrast, the incidence of hypothermia in the enhanced warming group showed a decreasing trend, dropping from 31.91 to 27.66%.

### 3.3 Coagulation parameters

Coagulation parameters at each time point are summarized in Table 2. No significant between-group differences in were observed for PT or INR at any time point (*p* > 0.05). For PT%, no significant between-group differences were found at C1 or C2, but the enhanced warming group showed a significantly higher PT% than the routine warming group at C3 (*p* = 0.049). For APTT, no significant between-group differences were observed at C1 or C2 (*p* > 0.05), whereas the enhanced warming group had significantly lower APTT than the routine warming group at C3 (*p =* 0.018). Within-group analyses showed that APTT at C3 was significantly prolonged compared to C1 in both groups (routine group, *p* < 0.01; enhanced group, *p* < 0.05). TT showed no between-group differences at all time points (*p* > 0.05).

**Table 2:** Coagulation parameters at different time points.

Parameter	Time point	Routine warming group (*n* = 87)	Enhanced warming group (*n* = 47)	U value	*P*-value
PT (s)	C1	14.70 (12.50, 25.30)	14.40 (12.00, 60.40)	1,830	0.318
	C2	15.70 (13.00, 69.40)	14.85 (12.70, 68.40)	1,580	0.059
	C3	14.90 (11.70, 47.00)	14.60 (12.00, 36.50)	1,616	0.07
PT% (%)	C1	78.30 (56.00, 105.00)	82.23 (48.00, 109.00)	1,780	0.259
	C2	70.00 (9.00, 105.00)	78.50 (10.00, 109.00)	1,572	0.054
	C3	76.00 (14.00, 140.00)	82.00 (19.00, 129.00)*	1,583	0.049
INR	C1	1.13 (0.93, 2.31)	1.11 (0.88, 6.78)	1835	0.33
	C2	1.24 (0.85, 8.48)	1.16 (0.95, 7.93)	1,599	0.072
	C3	1.17 (0.85, 5.12)	1.13 (0.88, 3.81)	1,593	0.054
APTT (s)	C1	35.30 (25.50, 96.30)	35.30 (26.00, 143.20)	1,634	0.056
	C2	37.60 (25.50, 180.00)	35.90 (28.70, 180.00)	1,613	0.083
	C3	38.70 (27.70, 180.00)**	36.80 (29.20, 180.00)*	1,503	0.018
TT (s)	C1	17.10 (13.80, 60.70)	17.60 (14.40, 48.10)	1,634	0.056
	C2	16.40 (13.50, 197.90)	16.90 (14.00, 112.20)	1,572	0.054
	C3	16.10 (13.90, 240.00)**	15.80 (15.40, 240.00)**	1,954	0.838
Fibrinogen (g/L)	C1	3.00 (0.60, 7.88)	2.70 (0.60, 7.13)	1,569	0.026
	C2	2.87 (0.60, 8.34)	2.09 (0.60, 6.97)	1,432	0.009
	C3	3.68 (0.60, 9.53)***	3.11 (0.60, 7.94)**	1,436	0.007
D-D (μg/L)	C1	10,560 (800, 20,000)	12,830 (190, 20,000)	1,963	0.7
	C2	11,330 (1,040, 20,000)	8,920 (280, 20,000)	1,793	0.444
	C3	6,650 (520, 20,000)***	6,360 (670, 20,000)***	1,989	0.969

Data presented as median (Q1, Q3). Between-group comparisons by Mann–Whitney U test. Within-group comparisons (vs. C1) by Wilcoxon signed-rank test: * *p* < 0.05, ** *p* < 0.01, *** *p* < 0.001. C1, 1 h preoperative; C2, 2–3 h postoperative; C3, 12–24 h postoperative. PT, prothrombin time; PT%, prothrombin time percentage; INR, international normalized ratio; APTT, activated partial thromboplastin time; TT, thrombin time; D-D, D-dimer.

Notably, fibrinogen in the enhanced warming group was significantly lower than that in the routine warming group at all three time points (C1, *p* = 0.026; C2, *p* = 0.009; C3, *p* = 0.007), including the preoperative baseline. Both groups showed significant increases in fibrinogen at C3 compared to C1 (both *p* < 0.001). D-dimer showed no significant between-group differences at any time points (*p* > 0.05). Within-group analyses showed significant decreases in D-dimer at C3 compared to C1 in both groups (*p* < 0.001).

### 3.4 Clinical outcomes

Clinical outcomes are presented in Table 3. The median length of hospital stay in the enhanced warming group was significantly shorter than that in the routine warming group (13.50 vs. 16.00 days, *p* = 0.040). No significant difference was observed in the incidence of postoperative complications between the two groups (*p* = 0.868).

**Table 3:** Recovery outcomes.

Outcome	Routine warming group (*n* = 87)	Enhanced warming group (*n* = 47)	*U/χ^2^* value	*P*-value
Length of hospital stay (d)^a^	16.00 (6.00, 56.00)	13.50 (3.00, 32.00)	*U* = 1,180	0.040
Postoperative complications^b^, *n* (%)	75 (86.21)	41 (87.23)	*χ^2^* = 0.028	0.868

a Presented as median (Q1, Q3), compared using Mann–Whitney U test.

b Postoperative complications were defined as the occurrence of one or more of the following within the hospital stay: surgical site infection, pneumonia, urinary tract infection, venous thromboembolism, reoperation for bleeding, organ dysfunction, or ICU readmission.

### 3.5 Multivariable regression analysis

To adjust for potential confounding, multivariable regression analyses were performed for the primary and secondary outcomes, with results presented in Table 4. After adjusting for age, sex, ISS, operative duration, intraoperative blood loss, and RBC transfusion volume, the enhanced warming strategy remained independently associated with a significantly lower risk of hypothermia at T3 (adjusted OR = 0.37, 95% CI 0.16–0.84, *p* = 0.018). In addition, longer operative duration was identified as an independent risk factor for hypothermia at T3 (adjusted OR = 1.006 per minute, 95% CI 1.001–1.012, *p* = 0.028).

**Table 4:** Multivariable regression analysis of the association between warming strategy and outcomes.

Outcome	Effect estimate^†^	95% CI	*P*-value
Hypothermia at T3 (primary)	aOR = 0.37	0.16–0.84	0.018
APTT at C3 (s)	*β* = −4.62	−8.87 to −0.37	0.033
PT% at C3 (%)	*β* = 4.83	−0.52 to 10.18	0.076
Length of hospital stay (d)	*β* = −3.42	−6.61 to −0.23	0.036

^†^Enhanced warming group as the exposure of interest (reference: routine warming group). All models adjusted for age, sex, ISS, operative duration, intraoperative blood loss, and RBC transfusion volume. The primary outcome was analyzed using multivariable logistic regression (Hosmer-Lemeshow goodness-of-fit *p* = 0.624); secondary outcomes were analyzed using multivariable linear regression. aOR, adjusted odds ratio; *β*, unstandardized regression coefficient; CI, confidence interval.

For coagulation parameters, the enhanced warming group showed a significantly lower APTT at C3 after adjustment (*β* = −4.62, 95% CI − 8.87 to −0.37, *p* = 0.033). The between-group difference in PT% at C3 did not reach statistical significance after multivariable adjustment (*β* = 4.83, 95% CI − 0.52 to 10.18, *p* = 0.076), suggesting that the univariate finding (*p* = 0.049) may have been influenced by confounding factors.

Regarding clinical outcomes, the enhanced warming strategy was independently associated with a shorter length of hospital stay (*β* = −3.42 days, 95% CI − 6.61 to −0.23, *p* = 0.036). ISS and intraoperative blood loss were also identified as independent predictors of prolonged hospitalization.

## 4 Discussion

This study compared the effects of routine and enhanced intraoperative warming strategies on perioperative hypothermia, coagulation function, and length of hospital stay in severely injured emergency surgery patients. The enhanced warming group in this study showed significantly higher core body temperature at end of surgery and 1 h postoperative, with a markedly lower incidence of hypothermia at T3 compared to the routine warming group. The routine warming group exhibited a substantial rise in hypothermia incidence from preoperative to postoperative time points, whereas the enhanced warming group showed a decreasing trend over the same interval. After adjusting for age, sex, ISS, operative duration, intraoperative blood loss, and RBC transfusion volume, the enhanced warming strategy remained independently associated with a reduced risk of postoperative hypothermia (adjusted OR = 0.37), supporting the possibility that the temperature benefit was not merely an artifact of baseline imbalances. These findings are consistent with prior evidence that forced-air warming devices can effectively reduce convective and evaporative heat loss by maintaining an elevated skin-surface microenvironment (19). Another previous study also demonstrated that warming intravenous fluids to near body temperature attenuates the thermal burden of large-volume resuscitation (20). The application of these methods, together with an elevated ambient operating room temperature, likely accounts for the different temperature trajectories observed between the two groups. A recent meta-analysis confirmed that forced-air warming during surgery significantly increases core body temperature and reduces hypothermia incidence, supporting the effectiveness of this approach even in prolonged procedures (21).

The multivariable analysis also identified operative duration as an independent risk factor for hypothermia at T3. This observation aligns with the understanding that prolonged surgical exposure amplifies cumulative heat loss through radiation, convection, and evaporation from exposed body surfaces and open surgical fields (22). In the present study, the median operative duration exceeded 170 min in both groups, which is considerably longer than that in typical elective surgical populations. Wang et al. (23) noted that duration of anesthesia or surgery is among the top five risk factors for perioperative hypothermia. Trauma patients undergoing lengthy emergency operations may be particularly vulnerable due to the combination of large wound areas, ongoing hemorrhage, and aggressive fluid resuscitation (3). This finding suggests the need for temperature monitoring and proactive warming measures throughout extended emergency procedures.

Regarding coagulation parameters, the enhanced warming group showed a significantly lower APTT at C3 after multivariable adjustment, suggesting that maintaining normothermia may help preserve the intrinsic coagulation pathway in trauma patients. The APTT is particularly sensitive to temperature-dependent reductions in coagulation factor activity, as the intrinsic pathway involves a larger number of enzymatic steps, each of which is subject to thermal deceleration (18, 24). In contrast, the between-group difference in PT% at C3 did not retain statistical significance after controlling for confounders, indicating that the unadjusted finding may have been influenced by other perioperative variables. This discrepancy between APTT and PT% may reflect the differential temperature sensitivity of the intrinsic and extrinsic coagulation pathways. Hui et al. (25) demonstrated using thromboelastography that mild hypothermia impairs clot formation through mechanisms not fully captured by conventional coagulation assays such as PT and APTT, suggesting that the coagulation benefits of normothermia may extend beyond what standard laboratory tests can detect. A notable and unexpected finding in this study is that fibrinogen levels were significantly lower in the enhanced warming group across all time points, including the preoperative baseline. Because this difference was present before any warming intervention was applied, it cannot be attributed to the warming strategy itself. Instead, several alternative explanations should be considered. Although ISS, trauma type, and intraoperative blood loss did not differ significantly between the two groups, the relatively small sample size may have been insufficient to achieve balance on all clinically relevant variables. The enhanced warming group had a numerically higher median ISS and greater median blood loss, which, even without reaching statistical significance, could reflect a subpopulation with more pronounced consumptive coagulopathy (26). In trauma-induced coagulopathy, fibrinogen is the first coagulation factor to reach critically low levels following massive hemorrhage, and its depletion is driven by a combination of consumption at injury sites, hemodilution from fluid resuscitation, and hyperfibrinolysis (27, 28). The slightly higher proportion of abdominal trauma in the enhanced warming group may also be relevant, as abdominal injuries are frequently associated with greater visceral hemorrhage and consumptive fibrinogen loss (18). This finding underscores the importance of adjusting for pre-existing fibrinogen differences when evaluating the coagulation effects of perioperative warming strategies. Future prospective studies should include fibrinogen as a stratification variable at enrollment to minimize such confounding.

The enhanced warming strategy was also associated with a shorter length of hospital stay after adjusting for confounders. While this association is consistent with the broader literature linking perioperative normothermia to improved recovery (6, 29), the mechanisms underlying this relationship in trauma patients are likely to be indirect and multifactorial. Maintaining normal body temperature may facilitate coagulation homeostasis and reduce the need for additional hemostatic interventions, thereby enabling earlier mobilization (18). Normothermia also helps preserve immune function and protein synthesis, which are critical for wound healing and resistance to surgical site infections (30). Wang et al. (31) reported that hypothermia was an independent risk factor for prolonged ICU stay in a surgical cohort, further supporting the link between temperature management and recovery trajectories. However, given the retrospective nature of this study, a causal relationship between the warming strategy and shortened hospitalization cannot be established; the observed association may also reflect other unmeasured differences between the two treatment periods.

This study has several limitations. The retrospective, single-center design and the relatively small sample size, especially with only 47 cases in the enhanced warming group, limit statistical power and the generalizability of the findings. Although multivariable regression was used to adjust for key confounders residual confounding from unmeasured variables (such as severity of physiological derangement at presentation, time from injury to surgery, and specific surgical procedures performed) cannot be excluded. The natural grouping based on a practice change introduces the possibility of temporal confounding, as the two groups were drawn from different calendar periods during which other aspects of clinical care may also have evolved. The lack of randomization and blinding may have introduced measurement and ascertainment bias, as clinicians aware of the warming protocol in use could have influenced documentation or clinical decision-making. Additionally, this study did not analyze the effects of different trauma types and severity levels on warming effectiveness, nor did it include long-term prognostic indicators. Furthermore, plasmatic-based coagulation tests such as PT and APTT cannot fully reflect *in vivo* coagulation function, as they do not account for cellular components of hemostasis; future studies incorporating viscoelastic coagulation testing (e.g., thromboelastography) and platelet function testing would be needed to comprehensively assess the impact of hypothermia on coagulation, given that platelet aggregation can also be severely impaired by hypothermia. Finally, although core body temperature was monitored using esophageal or nasopharyngeal probes, differences between monitoring sites may affect the precision of temperature measurements (32).

## 5 Conclusion

In this retrospective cohort of severely injured emergency surgery patients, the enhanced warming strategy was associated with a lower incidence of postoperative hypothermia, reduced APTT, and shorter hospital stay after adjusting for potential confounders, while the between-group difference in PT% did not persist after adjustment and the lower fibrinogen levels in the enhanced warming group appeared to reflect pre-existing baseline heterogeneity. These findings suggest a potential benefit of enhanced intraoperative warming for perioperative temperature and coagulation management in trauma patients, although the retrospective, observational design precludes causal inference. Prospective, multicenter trials with pre-specified stratification for baseline coagulation status are needed to validate these results.

## Data Availability

The original contributions presented in the study are included in the article/supplementary material, further inquiries can be directed to the corresponding author.
